# Predictive Coding or Evidence Accumulation? False Inference and Neuronal Fluctuations

**DOI:** 10.1371/journal.pone.0009926

**Published:** 2010-03-29

**Authors:** Guido Hesselmann, Sepideh Sadaghiani, Karl J. Friston, Andreas Kleinschmidt

**Affiliations:** 1 Cognitive Neuroimaging Unit, Institut National de la Santé et de la Recherche Médicale, Gif sur Yvette, France; 2 Institut d'Imagerie Biomédicale NeuroSpin, Commissariat à l'Energie Atomique - Direction des Sciences du Vivant, Gif sur Yvette, France; 3 Department of Neurobiology, Weizmann Institute of Science, Rehovot, Israel; 4 International Max Planck Research School of Neural and Behavioural Sciences, University of Tübingen, Tübingen, Germany; 5 Wellcome Trust Centre for Neuroimaging, University College London, London, United Kingdom; Kyushu University, Japan

## Abstract

Perceptual decisions can be made when sensory input affords an inference about what generated that input. Here, we report findings from two independent perceptual experiments conducted during functional magnetic resonance imaging (fMRI) with a sparse event-related design. The first experiment, in the visual modality, involved forced-choice discrimination of coherence in random dot kinematograms that contained either subliminal or periliminal motion coherence. The second experiment, in the auditory domain, involved free response detection of (non-semantic) near-threshold acoustic stimuli. We analysed fluctuations in ongoing neural activity, as indexed by fMRI, and found that neuronal activity in sensory areas (extrastriate visual and early auditory cortex) biases perceptual decisions towards correct inference and not towards a specific percept. Hits (detection of near-threshold stimuli) were preceded by significantly higher activity than both misses of identical stimuli or false alarms, in which percepts arise in the absence of appropriate sensory input. In accord with predictive coding models and the free-energy principle, this observation suggests that cortical activity in sensory brain areas reflects the precision of prediction errors and not just the sensory evidence or prediction errors *per se*.

## Introduction

The notion that perception involves inference dates back for centuries and has been refined using mathematical models, grounded mostly in a Bayesian framework [1]. Yet, contemporary models of perceptual decisions differ in terms of neuronal implementation, and the neurophysiological evidence garnered in their support [2], [3]. In some accounts, cortical activity reflects sensory evidence that is accumulated to a critical level to yield a perceptual decision. This family of diffusion or race models can be regarded as dynamic extensions of signal detection theory [4]. In these models, the implicit neuronal code is a log-probability or likelihood-ratio code. In other hierarchical models, cortical activity encodes top-down predictions and bottom-up prediction error [5], [6]. The error signal is accumulated and used to optimise predictions and suppress prediction error or free-energy. In this case, inference rests on predictions that serve to explain away the difference between predicted and incoming sensory information.

Both views are supported by studies of evoked cortical responses during perceptual decisions [7], [8], [9]. However, it is difficult to say which model better explains empirical observations, because both can be formulated to give similar predictions. Recently, it has been shown that ongoing cortical activity, prior to sensory stimulation, can predict subsequent perceptual decisions [10], [11], [12], [13]. As ongoing activity fluctuates between trials, so does the perception of identical stimuli. Critically, the two theoretical accounts make qualitatively different predictions about the relationship between ongoing activity and perception. Put simply, under evidence accumulation models, activity increases with the evidence for a stimulus, whereas under predictive coding it reflects the precision of the prediction error [14]. Therefore, in evidence accumulation models, high ongoing activity will bias inference to detection with (true hits) or without (false alarms) an appropriate stimulus. Conversely, under predictive coding, ongoing activity levels in sensory cortex reflect the precision (inverse variance) of sensory noise. When sensory noise is low prediction errors are amplified. If sensory noise is high, this induces self-inhibition among units coding prediction error and leads to a relative increase in the influence of top-down predictions [14]. In this setting, false alarms are emitted when the precision is too low to counter top-down predictions for which there is no sensory evidence.

In short, accumulation models suggest high ongoing activity will bias towards stimulus detection (true hits or false alarms). Conversely, the predictive coding or free-energy formulation suggests that high ongoing activity (i.e., precise prediction errors) will bias towards correct inference (hits or correct rejections). This means we can adjudicate between the two models by examining pre-stimulus activity for hits, correct rejections, false alarms and misses.

Here, we report functional magnetic resonance imaging findings related to false alarms in two perceptual paradigms. Findings from both experiments have been reported previously but only with respect to hits and misses (where we had greater trial numbers) [12], [13]. However, these two conditions alone do not permit any conclusion regarding the nature of the signal, prediction error or sensory evidence. We therefore conducted a new analysis that included those subjects in the two experiments with a sufficient number of false alarms for statistical analysis. We obtained small but significant effects that were consistent across both experiments and that suggest that neural activity in sensory areas codes the precision of prediction error.

## Results

**Figure 1:**
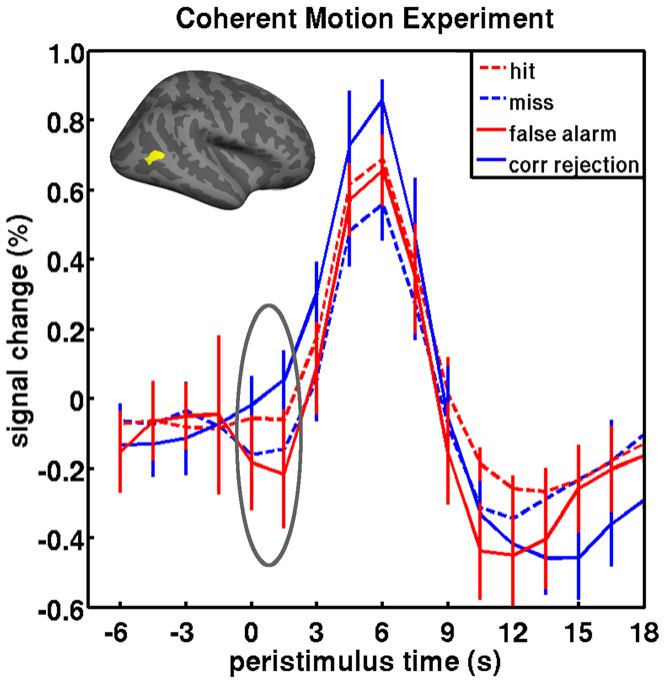
Peristimulus fMRI signal time-courses from the visual motion experiment. Data were normalized to grand mean and averaged across 9 subjects (bars represent standard error of the mean) performing a motion coherence judgment task. The insert specifies the conditions as a function of stimulus and percept. The inflated right hemisphere rendering of the group result shows the right hMT+ region of interest, which was identified subject by subject in a localizer procedure employing coherent motion stimuli vs. static displays. The grey ellipse covers the pre-stimulus period submitted to statistical testing (see main text).

The first experiment involved detecting motion coherence in random dot kinematograms with coherent motion at threshold (periliminal) in most trials and above or below threshold (supra- and subliminal) in a smaller number of trials [12]. We measured cortical activity, prior to evoked responses (grey ellipse in Fig. 1), in the human visual motion complex V5/hMT+. According to accumulation models we should observe pre-stimulus activity levels for: hits and false alarms > misses and correct rejections. And, according to predictive coding: correct rejections and hits > misses and false alarms. Our empirical observations confirmed the latter (Fig. 1).

The greatest difference in pre-stimulus activity was between the correct rejections and false alarms (solid blue and red lines, respectively). This is clear evidence that pre-stimulus activity reflects the precision (predictive coding) of the subsequent percept not its content (evidence accumulation). More formally, an ANOVA of the differences across activity at time points 0 and 1.5 s showed a main effect of accuracy, correct vs. incorrect (p<.022, consistent with predictive coding), but no main effect of percept, coherent vs. incoherent (predicted by accumulation). In *post-hoc* t-tests, pre-stimulus activity in subsequent hit-trials was significantly greater than misses; and activity in false alarms were significantly less than in correct rejects (p = .048 and p = .031, respectively, unpaired one-sided t-tests; on a qualitative level, “hit>miss” in 8/9 subjects and “correct rejection>false alarm” in 6/9). The use of one-sided *post-hoc* t-tests was justified by the directed assumptions of the two models that we considered; accumulation vs. prediction. It should be pointed out that these effects were not significant when just testing activity in a single epoch (0 or 1.5 s) as in our previous analyses of hits and misses alone. This observation indicates a loss of statistical power relative to previous analyses that included a greater number of subjects [12].

To determine the topographic specificity of the observed effects, we analyzed BOLD time courses in a set of control regions that were robustly activated or deactivated by the motion task. These regions included areas involved in early visual motion processing (V1/V2), as well as attention and perceptual decision making (right IPL, right and left FEF, right IFG, and ACC). No region showed the “hit>miss” or “correct rejection>false alarm” effects in the pre-stimulus baseline, and subsequent voxel-based whole brain analyses were also negative.

In the second experiment, we studied detection of auditory signals presented at threshold against ongoing scanner noise [13]. This detection paradigm can be reconciled with the form of the previous experiment by regarding it as a continuous discrimination, with two alternatives of stimulus ‘present’ or ‘absent’. However, this free-response paradigm does not furnish correct rejection trials (i.e., subjects are not required to indicate the stimulus is absent). We expected the difference between hits and false alarms to be even more pronounced than in the first experiment. This is because in the auditory fMRI experiment ongoing sensory noise levels were higher due to scanner noise than in the visual experiment, where inter-stimulus intervals contained a stationary dot pattern. Under predictive coding, this higher sensory noise should suppress the gain of error units and reduce activity levels, accentuating the effect of endogenous fluctuations.

**Figure 2:**
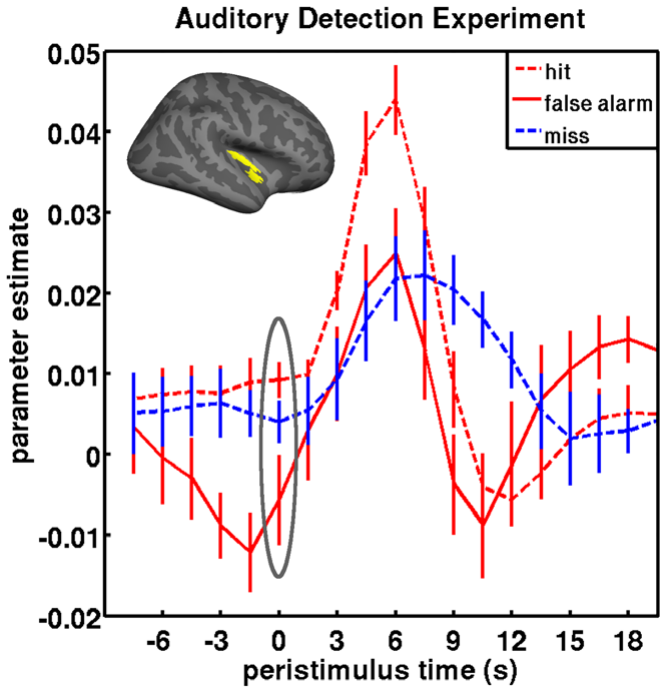
Peristimulus fMRI signal time-courses from the auditory experiment. Data were estimated under a finite response model and averaged across 9 subjects (bars represent standard error of the mean) performing an auditory stimulus detection task. Data are plotted for conditions specified by an insert. The inflated right hemisphere rendering of the group result shows the location of the region of interest, which includes early auditory cortex with parts of Heschl's gyrus (identified bilaterally subject by subject). The grey ellipse covers the pre-stimulus period submitted to statistical testing (see main text).

As before, the predictions of the two theoretical accounts differ: Evidence accumulation would expect hits and false alarms (i.e., an auditory percept) to follow higher baseline levels, relative to misses (no percept). Conversely, the predictive coding account suggests that (incorrect) misses and false alarms are foreshadowed by significantly lower activity than (correct) hits. Our findings in this experiment supported the latter prediction (Fig. 2). False inference (false alarms - red solid lines, and misses - blue dashed lines) were preceded by significantly lower levels of activity in auditory cortex than veridical hits (red dashed line, p = .021 and p = .018, respectively, in unpaired one-sided t-tests; on a qualitative level, “hit>miss” in 7/9 subjects and “hit>false alarm” in 7/9). There was no significant difference in activity preceding misses vs. false alarms. This low activity prior to false alarms is consistent with a scheme that under-weights sensory evidence via an inhibition of error units and thus fails to constrain top-down predictions. The profound activity dip in the 3 s preceding a false alarm could indicate a critical level of ongoing activity that is necessary for the endogenous generation of a percept, in the absence of the stimulus. Conversely, in the case of misses, it is the dwindling of a prediction (or the toggling to the alternative prediction) and the associated increase in the noise estimation for top down influences that via self-inhibition down-regulates the local fMRI signal such that despite sensory input no percept is reported.

In contrast to our local findings in the motion experiment, the pre-stimulus effects in the auditory experiment appeared to be much less spatially confined. Similar to the original publication–where we report distributed “hit>miss” and “miss>hit” effects -, maps (at t = 0 s) of “hit>false alarm” show a number of regions outside of auditory cortex, including mid-cingulate cortex, polar and ventro-medial prefrontal cortex and early visual cortex (cluster-level *p*<0.05, corrected, after auxiliary *p*<0.005, uncorrected). The reverse contrast “false alarm>hit” yielded no significant foci (p<0.05, uncorrected). Note that such a difference between the experiments was to be expected because the auditory paradigm involved detection of near-threshold stimuli in a free-response setting instead of two-alternative forced choice decisions on ambiguous but clearly notable stimulation in the motion experiment.

In both experiments, we found no relationship between the duration of the prior SOA and behavioural outcome. In the motion experiment, the SOAs were 29.2±0.8 s for hits, 30.3±0.9 s for misses, 29.6±1.3 s for correct rejections, and 30.1±3.2 s for false alarms (mean ± sd). In the auditory experiment, the SOAs were 30.6±0.2 s for hits, and 30.4±0.4 s for misses. False alarms occurred 17.8±1.1 s after the preceding stimulus, i.e. approximately in the middle between two auditory stimuli.

## Discussion

Both experiments support an interpretation of neural activity (indexed by fMRI signal) in specialized sensory cortical regions as coding prediction error and not evidence or log-probability (cf. classical signal detection theory). Our analyses were conducted using the responses of brain regions that are specialised for the sensory information required for the subjects' perceptual decisions. Our findings therefore cannot be compared with those obtained in higher order (polymodal) cortex like the lateral intra-parietal and premotor areas. However, our findings can be compared to studies of sensory cortex, where baseline variations were removed [7].

In terms of neuronal computation, the free-energy principle encompasses evidence accumulation schemes as a special case that is manifest at higher levels in the sensory hierarchies, as prediction error is accumulated to optimise high-level representations and the ensuing top-down predictions of sensory input [15]. The evidence we present here in favour of the free-energy principle comes from sensory regions and from the analysis of perceptual outcome as a function of activity prior to stimulation. Our analysis was constrained to pre-stimulus windows, because this avoids the confounding effect of evoked signal changes (e.g., differences in sensory stimulus properties and their frequency as well as their perception and behavioural consequences). Yet, the same principle is likely to hold throughout the entire time series of neural activity. For instance, the evoked responses in the first experiment show a main effect of stimulus type with greater responses to incoherent motion stimuli. Again, this argues against a coding of sensory evidence and in favour of a coding of the greater “surprise” associated with the less frequent incoherent motion stimuli, compared to the more frequent periliminal coherent motion stimuli. It is also obvious from our findings that widely applied analysis features such as baseline normalisation to pre-stimulus signal may distort effects observed in the evoked responses.

Our study departs from usual treatments of neuroimaging results in terms of predictive coding [16] because we did not look for the correlates of prediction error; we tried to disambiguate between evidence accumulation and predictive coding schemes. This means we had to dissociate the effects of precision and prediction error *per se* (which are conflated during the expression of precision-weighted prediction error). We therefore focussed on pre-stimulus activity levels, which can only reflect putative changes in the precision that is conferred on prediction errors, when they are later induced by a stimulus.

An important limitation of our analyses is that functional neuroimaging, while useful in recording population synaptic activity, does not resolve the fast dynamics underpinning perceptual decisions. This limitation is tempered by previous functional neuroimaging studies, where fluctuations in ongoing activity can predict subsequent percepts on a trial by trial basis [11], [12], [13]. In other words, we can exploit fluctuations in neuronal activity and subsequent perceptual processing to establish causal relationships through temporal precedence, even with slow hemodynamic signals. As in previous studies, we analysed time segments of the signal that are as close to the upcoming stimulus as possible without including stimulus-driven responses We refer to the peri-stimulus fMRI responses until stimulus onset as ‘pre-stimulus’. However, because the hemodynamic response delays and disperses underlying neuronal activity, some ‘pre-stimulus’ neuronal activity will actually appear after stimulus onset in the fMRI time-series. Happily, the converse situation (post-stimulus neuronal activity confounding pre-stimulus fMRI data) cannot occur. We suppose that the fluctuations in baseline activity we recorded with fMRI reflect endogenous (ongoing) fluctuations in fast neuronal activity. Indeed, computational studies suggest that fast synchronised activity fluctuates in power with the characteristic ultra-slow frequencies seen in fMRI [17]. Furthermore, the general picture from combined EEG and fMRI studies [18] suggests that increases in fast oscillatory activity elevate BOLD signals. These slow modulations of fast activity are, we presume, mediated by neuromodulatory effects at a synaptic level. These effects underlie changes in post-synaptic gain of the sort associated with attention [19] and perceptual precision (i.e., signal to noise) [20].

Our findings illustrate that pre-stimulus fMRI signals cannot be interpreted as encoding sensory evidence but are consistent with an alternative explanation that it reflects the level of attention. Indeed, attention modulates cortical activity in sensory areas even in the absence of input [21]. This interpretation is not at odds with an account grounded in the free-energy principle, because the increased precision that may be reflected in higher levels of endogenous activity is thought to mediate the effects of directed attention [15]. This means that the optimization of precision in predictive coding and attention become the same thing. Whether this necessarily applies to our observations is a complicated issue. An effect of fluctuations on directed (endogenous) attention should fulfil more criteria than mere modulation of local signal in a sensory area. Among these are associated effects in higher order attentional control centres, increased sensory response amplitudes and shortened reaction times. No such evidence was found in our first experiment [12] and only partial support in the second experiment, where attention and awareness are not easily dissociated in response amplitudes and where reaction times are only available for hits [13]. Conversely, the insufficient evidence for an attention account does not invalidate the interpretation along the lines of the more general free-energy principle. In short, whatever the neural or cognitive origin of endogenous fluctuations, their impact on perceptual performance is captured by predictive coding models. This is an important conclusion because the functional role of such ongoing or endogenous activity fluctuations, which have traditionally been neglected in many neurophysiological and theoretical investigations of perceptual inference and decisions, is becoming increasingly evident [22], [23], [24], [25].

## Materials and Methods

### Ethics Statement

Both studies received ethics committee approval by the authorities responsible for our institution (INSERM-CEA, NeuroSpin). All subjects gave written informed consent.

### Data acquisition and pre-processing

Details of both experiments have been published previously [12], [13]. Imaging data for both studies were acquired on a 3T MRI scanner (Tim Trio, Siemens, Erlangen). Functional imaging used a T2*-weighted gradient-echo, echo-planar imaging sequence (25 slices, TR = 1500 ms, TE = 30 ms, FOV 192, voxel size 3×3×3 mm, inter-slice gap 20%). Anatomical imaging used a T1-weighted MPRAGE sequence (160 slices, TR = 2300 ms, TE = 2.98 ms, FOV 256, voxel size 1.0×1.0×1.1 mm for the motion experiment, and 176 slices, TR = 2300 ms, TE = 4.18 ms, FOV 256, voxel size 1×1×1 mm for the auditory experiment). We used SPM5 (http://www.fil.ion.ucl.ac.uk, Wellcome Trust Centre for Neuroimaging, London, UK) for image pre-processing that involved realignment, coregistration, normalization to MNI stereotactic space, spatial smoothing with an isotropic Gaussian kernel of 6 and 12 mm (motion experiment) or 5 and 6 mm (auditory experiment) full-width-half-maximum for single subject and group analyses, respectively and estimation of general linear models.

### Motion experiment

Twelve right-handed subjects with normal or corrected-to-normal visual acuity (6 female, ages 19–30) participated in the motion experiment. Stimuli were dynamic dot displays of 500 white squares (size 0.2°) randomly distributed on a dark grey annulus (23°). Subjects were instructed to maintain gaze within a central blue rectangle (1°) surrounded by a light grey circular patch (3°) throughout the experimental sessions. For 355 ms intervals, stimuli moved up- or downwards, at 14°/s and with variable motion coherence. Subjects were asked to report as quickly and accurately as possible by button presses after each stimulus whether they had perceived coherent or random motion. Prior to scanning we determined individual motion coherence thresholds based on the method of constant stimuli (average motion coherence threshold across subjects 13%, range 8 to 20%). During fMRI scanning, three motion coherence levels were used: subliminal (1% coherence, 20 trials), periliminal (individual threshold, 60 trials), and supraliminal (30% coherence, 20 trials). Stimuli were presented in two 25 minute runs with 50 trials each. Between stimuli, the display was static for inter-stimulus intervals (ISI) of 20 to 40 s that were randomly selected from a uniform distribution.

Functional images for two 1000 volume experimental runs and one 208 volume localizer run were acquired. Localizer fMRI runs identified cortical regions sensitive to two types of coherent visual motion, up- or downwards motion and an expanding ‘starfield’. Continuous 16 s motion blocks were separated by 10 s stationary periods, and each condition was repeated over 6 blocks in counter-balanced order. Motion-sensitive areas were identified by mapping for each subject the contrast ‘motion > stationary’ at p<0.001, uncorrected. A local maximum near the ascending limb of the inferior temporal sulcus was defined as hMT+ (see original publication for coordinates). After removing session effects and linear trends from the BOLD signal time series of the main experiment, we extracted the percent signal change time courses of all periliminal and subliminal trials from 4 scans (6 s) before to 12 scans (18 s) after target onset and sorted them according to hits (perceiving periliminal stimuli as coherent), misses (periliminal stimuli as random), correct rejections (subliminal stimuli as random), and false alarms (subliminal stimuli as coherent). Here, we only report data from those subjects who generated a sufficient number of false alarms (n = 9 out of 12 subjects). Across subjects, near-threshold stimuli generated 57% hits and 43% misses, and subliminal stimuli 74% correct rejects and 26% false alarms.

### Auditory experiment

Twelve right-handed normal hearing subjects (2 female; ages 19–30) participated in the auditory experiment. One subject reported to have fallen asleep in one session and was thus excluded from analysis. Subjects were exposed to sparse near-threshold auditory stimuli and performed an auditory detection task. The stimulus was a 500 ms noise burst with its frequency band modulated at 2 Hz (from white noise to a narrower band of 0–5 kHz and back to white noise). Subjects were blindfolded and instructed to report as quickly and accurately as possible by a right hand key press whenever they heard the target sound despite scanner's background noise. In a first 6.5 min fMRI run, which was not analyzed, we determined each subject's auditory threshold using a simple staircase procedure with 25 trials and inter-stimulus intervals randomized between 2.5 and 5 s. Next, each subject performed 2 and some subjects 3 experimental runs of 20 min duration. In each run, target stimuli were presented at individual threshold (periliminal stimuli) on 36 trials and at a fixed supra-threshold level on 4 ‘catch’ trials. ISIs ranged unpredictably from 20 to 40 s, with each specific ISI used only once. Before each run, the target stimulus was played a few times at supra-threshold volume for (re)memorization and subjects were informed that in most of the trials the target sound would be played at a barely perceptible level. If within 1.5 s of stimulus onset a key was pressed this trial was counted as a hit, if not as a miss. All other key presses were classified as false alarms. Here, we restricted our analysis to those subjects who generated at least 5 false alarms (n = 9 out of 11). These subjects detected 59±17% of the near-threshold stimuli.

Experimental runs consisted of 820 volumes. An additional passive localizer run for defining auditory responsive brain regions was acquired after the main experiment. This 81 volume run consisted of three 20 s-blocks of repetitive stimulus presentation with 0.5 s inter-stimulus intervals (ISI) at clearly audible volume separated by 15 s baseline epochs. Voxels responding to the auditory stimulus were defined on a subject-by subject basis in two steps. First, at the group level the contrast periliminal stimuli (i.e. hits and misses) > baseline (p<0.001) was masked by the passive auditory localizer contrast at p<0.001. A spherical search space of 10 mm was defined around the peak of the peri-Heschl clusters with the highest z-score. Next, for each subject's corresponding first-level contrast all voxels within this search space were selected that passed a lenient threshold (p<0.05, uncorrected).

As false alarms occurred at unpredictable times and sometimes prior to hemodynamic relaxation, we could not directly analyze fMRI signal time courses as in the motion experiment. We hence used a finite impulse response (FIR) model on the high-pass filtered data with very low cutoff (1/1000 Hz) and no pre-whitening to ensure linear drift removal while minimizing interference with low frequency brain activity fluctuations. The FIR model used 24 peristimulus stick functions (x 1.5 s bins) for each of the four conditions, hits, misses, false alarms and catch trials. For near-threshold stimuli (hits and misses) time-locking was based on stimulus presentation; for false alarms it was based on behavioural reports minus the subject's average reaction time in hit trials (794±109 ms).
